# Curcumin Improves the Tumoricidal Effect of Mitomycin C by Suppressing ABCG2 Expression in Stem Cell-Like Breast Cancer Cells

**DOI:** 10.1371/journal.pone.0136694

**Published:** 2015-08-25

**Authors:** Qianmei Zhou, Meina Ye, Yiyu Lu, Hui Zhang, Qilong Chen, Shuang Huang, Shibing Su

**Affiliations:** 1 Research Center for Traditional Chinese Medicine Complexity System, Shanghai University of Traditional Chinese Medicine, Shanghai, China; 2 Longhua hospital, Shanghai University of Traditional Chinese Medicine, Shanghai, China; 3 Department of Biochemistry and Molecular Biology, Medical College of Georgia, Augusta, GA, United States of America; University of Navarra, SPAIN

## Abstract

Cancer cells with stem cell–like properties contribute to the development of resistance to chemotherapy and eventually to tumor relapses. The current study investigated the potential of curcumin to reduce breast cancer stem cell (BCSC) population for sensitizing breast cancer cells to mitomycin C (MMC) both in vitro and in vivo. Curcumin improved the sensitivity of paclitaxel, cisplatin, and doxorubicin in breast cancer cell lines MCF-7 and MDA-MB-231, as shown by the more than 2-fold decrease in the half-maximal inhibitory concentration of these chemotherapeutic agents. In addition, curcumin sensitized the BCSCs of MCF-7 and MDA-MB-231 to MMC by 5- and 15-fold, respectively. The BCSCs could not grow to the fifth generation in the presence of curcumin and MMC. MMC or curcumin alone only marginally reduced the BCSC population in the mammospheres; however, together, they reduced the BCSC population in CD44^+^CD24^−/low^ cells by more than 75% (29.34% to 6.86%). Curcumin sensitized BCSCs through a reduction in the expression of ATP-binding cassette (ABC) transporters ABCG2 and ABCC1. We demonstrated that fumitremorgin C, a selective ABCG2 inhibitor, reduced BCSC survival to a similar degree as curcumin did. Curcumin sensitized breast cancer cells to chemotherapeutic drugs by reducing the BCSC population mainly through a reduction in the expression of ABCG2.

## Introduction

Cancer stem cells were first discovered in acute myelogenous leukemia in 1994 [1,2]. However, the importance of cancer stem cells in tumorigeneity was investigated only in 2003, after the discovery of the first solid tumor stem cells in breast cancer stem cells (BCSCs). BCSCs expressing CD44^+^CD24^−/low^ surface markers isolated from human breast cancer clinical specimens were found to be highly tumorigenic [3]. Sphere culture is currently the most common method [4] for enriching a CD44^+^CD24^−/low^ breast cancer cell population by 40–98% from clinical specimens or cell lines [5,6].

Cancer stem cells, akin to other stem cells, can self-renew and differentiate. In addition, they display various phenotypes within the tumor, thus contributing to tumor heterogeneity [7]. Although cancer stem cells comprise only a minor population within the tumor, they are resistant to conventional chemotherapy and radiation therapy [8,9]. Moreover, administering chemotherapeutic agents can increase the population of cancer stem cells [10]. For example, human breast cancer cells implanted in epirubicin-treated mice were found to be considerably enriched with CD44^+^/CD24^−/low^ cells [11]. The chemoresistance observed in cancer stem cells is attributed to various mechanisms, such as their more effective DNA repair, reduced immunogenicity, inherent antiapoptotic properties, and quiescence [12]. However, It has shown that the overexpression and preferential activation of ATP-binding cassette (ABC) transporters are the major causes leading to cancer stem cell chemoresistance in 2013[13].

Multidrug-resistant (MDR) transporters are members of the ABC transporter superfamily and are prominent in cancer cell drug resistance [14]. Extensive studies have linked 3 ABC-superfamily multidrug efflux pumps, namely ABCB1/MDR1, ABCC1/MRP1, and ABCG2/BCRP, to cancer cell drug resistance [15]. The physiological role of ABCB1/MDR1 is to excrete toxic metabolites in normal tissue epithelium, including the kidneys, liver, intestine, pancreas, placenta, and adrenal gland [16]. However, ABCB1/MDR1 is expressed in various solid cancers and directly contributes to chemotherapy failure [17]. In addition to ABCB1/MDR1, ABCC1/MRP1 is overexpressed in several drug-resistant cancer cells and can confer resistance to several antitumor drugs, such as anthracyclines, vinca alkaloids, and camptothecins [18,19]. ABCG2/BCRP was initially isolated from drug-resistant breast cancer cells and is a key factor in determining drug absorption, distribution, and elimination [20]. Furthermore, recent studies have linked cancer stem cell chemoresistance to ABC transporters [21]. For example, the high drug-resistance of glioblastoma stem cells is mainly because of the enhanced expression of ABCB1. ABCG2 expression inhibition sensitizes liver cancer stem cells to chemotherapeutic agents. Because ABC transporters are highly expressed in cancer stem cells, approaches that target cancer stem cells by inhibiting ABC transporters have been devised.

Various antagonists of MDR efflux pumps, such as fucoxanthin and canthaxanthin, have recently been shown to reverse multidrug resistance in cancer cells by interfering with ABC transporters [22]. Fumitremorgin C, a highly specific ABCG2 inhibitor, is too neurotoxic for clinical use; despite their effectiveness in vitro, these inhibitors are scarcely suitable for clinical application for cancer treatment because of their intolerable toxicities. Thus, finding modulators against MDRs that are both effective and nontoxic is a major challenge [23].

In a previous study, we showed that curcumin improves the antitumor effect of MMC on breast cancer cells [24]. However, the mechanism associated with curcumin-mediated drug sensitization is unknown. Curcumin can inhibit the growth of cancer stem cell–initiating cells [25,26]. Thus, we hypothesize that curcumin-mediated chemosensitization is due to curcumin’s ability to target cancer stem cell–like cells. In this study, we showed that curcumin sensitized BCSCs to chemotherapeutic drugs both in vitro and in vivo by suppressing the function of ABCG2, thus improving the tumoricidal effect of chemotherapeutic drugs.

## Materials and Methods

### Cell culture, mammosphere-forming assay, and efficiency

Human breast cancer cell lines MDA-MB-231 and MCF-7 were purchased from American Type Culture Collection (Rockville, MD, USA) and cultured in Dubecco’s modified Eagle’s medium (DMEM) (Gibco, Scotland, UK) supplemented with 10% heat inactivated fetal bovine serum at 37°C in a humidified incubator supplied with 5% CO_2_. Mammospheres were generated by seeding MDA-MB-231 and MCF-7 cells at a density of 10^3^ cells/cm^2^ in 6-well ultralow attachment plates and a mammosphere culture medium (F-12/DMEM, 5 mg/mL insulin, 2% B27, 10 ng/mL basic fibroblast growth factor, and 20 ng/mL human recombinant epidermal growth factor). The mammosphere-forming efficiency (MSFE) was calculated as the percentage: [number of sphere-like structures (diameter >50 μm) formed in 7 d/original number of cells seeded]

### Flow cytometry analysis of BCSCs

MDA-MB-231 and MCF-7 cells were trypsinized to form a single cell suspension. Cells were labeled in the dark with CD44-FITC and CD24-PE or their respective isotype controls for 30 min. The labeled cells were washed to remove unbound antibodies, fixed in phosphate buffered saline containing 1% paraformaldehyde, and analyzed using a fluorescence activating cell sorter (Becton Dickinson, CA, USA) within 1 h of staining.

### Cell viability assay

BCSCs were plated onto 96-well plates in a stem cell culture medium containing various concentrations of drugs. Cell viability was assessed through a 3-(4,5-dimethylthiazol-2-yl)-2,5-diphenyltetrazolium bromide (MTT) assay (Promega, Madison, WI) as previously described [27]. Cytotoxicity was expressed as a percentage: (cells surviving/total number of untreated cells).

### Animal model establishment and drug intervention

Six-week-old female BALB/c nude mice were obtained from Academia Sinica (Shanghai, China) and housed under pathogen-free conditions in ventilated cages. All procedures were conducted with animal welfare considerations and were approved by the Ethical Committee of Shanghai University of Traditional Chinese Medicine. The tumorigenic potential of the mammospheres was assessed through their ability to generate tumors in nude mice. From spherical colonies, 100 000 dissociated cells were collected through magnetic-activated cell sorting based on CD44^+^CD24^−^ surface labeling, suspended in 200 μL of PBS, and inoculated into the mammary fat pad of the mice. Tumor development was monitored every 5 d for up to 5 wk and tumor volumes (V) were calculated using V = 1⁄2 × D_max_ × (D_min_)^2^, where D_max_ is the maximal tumor diameter and D_min_ is the corresponding perpendicular diameter [28].

### Statistical analyses

Statistical differences were determined using 2-tailed Student *t* test. Data are presented as mean ± standard deviation (n ≥ 3). Level of significance was set at *P* < 0.05.

## Results

### Curcumin increases the sensitivity of paclitaxel, cisplatin, and doxorubicin to breast cancer cells

**Fig 1:**
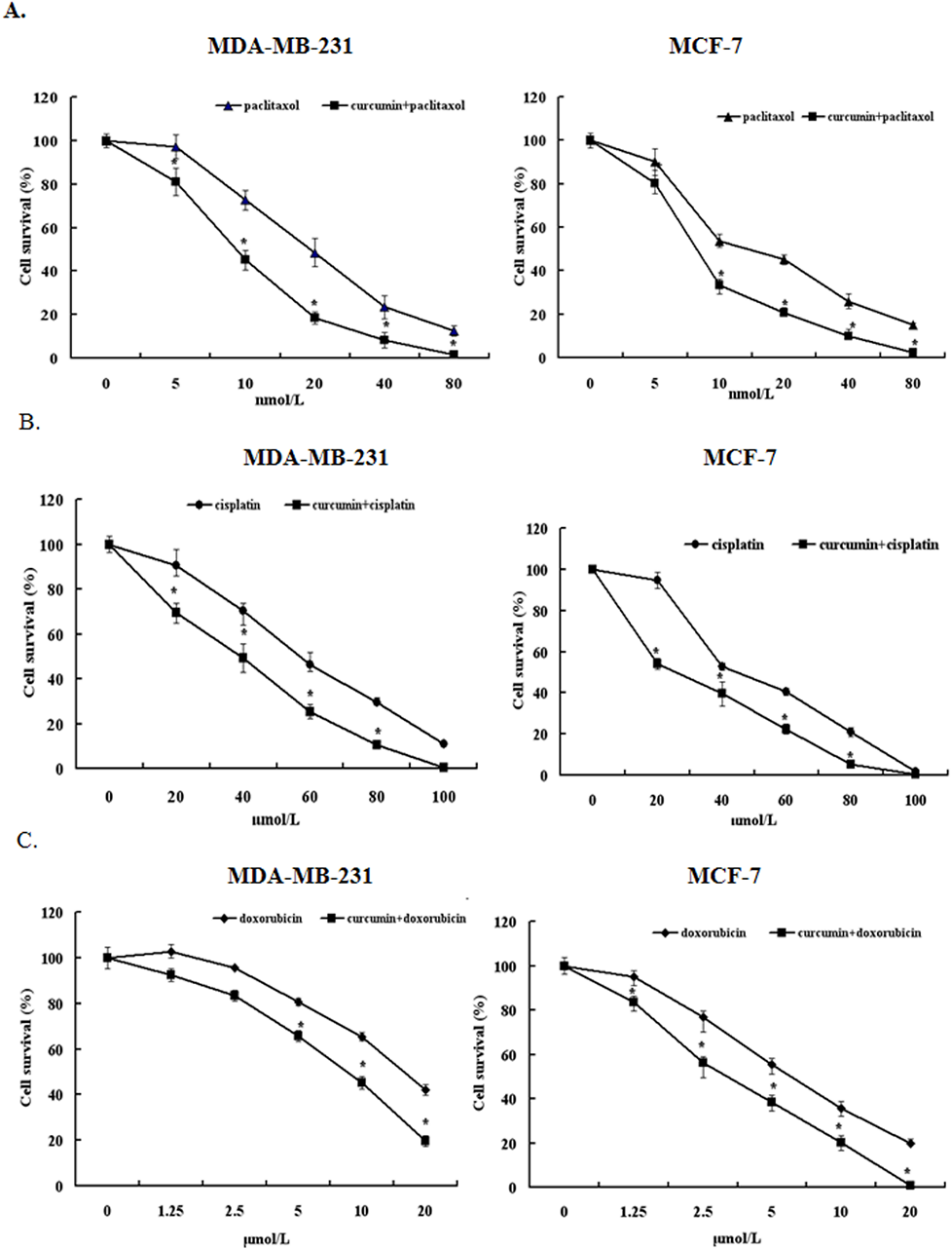
Curcumin and chemotherapeutic drugs (paclitaxol, cisplatin, and doxorubicin) alone and together inhibited the cell viability of MDA-MB-231 and MCF-7 cells. Cells were treated with different concentrations of paclitaxel, cisplatin, and doxorubicin and/or 40 μmol/L curcumin (IC_50_). An MTT assay was used to determine cell viability at 48 h. Data are presented as means ± SD (n = 3). *P < 0.05 and compared with untreated control.

We have previously reported that curcumin sensitizes breast cancer cells to MMC. To determine whether curcumin also improves the tumoricidal effect of other commonly used chemotherapeutic agents, we treated MDA-MB-231 and MCF-7 cells with paclitaxel, cisplatin, and doxorubicin in the presence and absence of curcumin. Curcumin reduced the half-maximal inhibitory concentration (IC_50_) of paclitaxel, cisplatin, and doxorubicin in MDA-MB-231 cells from 20 to 10 nmol/L, 60 to 40 μmol/L, and 15 to 7.5 μmol/L, respectively (Fig 1A, 1B and 1C). Similarly, curcumin significantly reduced the IC_50_ of paclitaxel, cisplatin, and doxorubicin in MCF-7 cells from 10 to 7.5 nmnol/L, 40 to 20 μmol/L and 5 to 3 μmol/L, respectively (Fig 1A, 1B and 1C). Thus, curcumin sensitized breast cancer cells to these commonly used chemotherapeutic agents.

### Curcumin enhances the capability of MMC to reduce BCSC population

Because the chemoresistance of cancer cells is closely associated with their stem cell–like nature, we investigated the possibility that curcumin confers chemosensitivity by regulating the BCSC population by establishing BCSCs from MCF-7 and MDA-MB-231 cells by culturing them in stem cell culture conditions. Floating spherical colony-like structures were observed 2 d after culturing in the 2 cell lines (S1 Fig). MDA-MB-231 cells grew to 100 μm in diameter in 1 wk; however, MCF-7 cells grew to only 50 μm in diameter in 1 wk (S2 Fig), indicating that MDA-MB-231 cells contain a larger population of stem cell–like cancer cells. Therefore, chemoresistance is functionally associated with the BCSC population, because MDA-MB-231 cells displayed higher IC_50_ than MCF-7 cells in the 4 tested chemotherapeutic agents (Fig 1).

**Fig 2:**
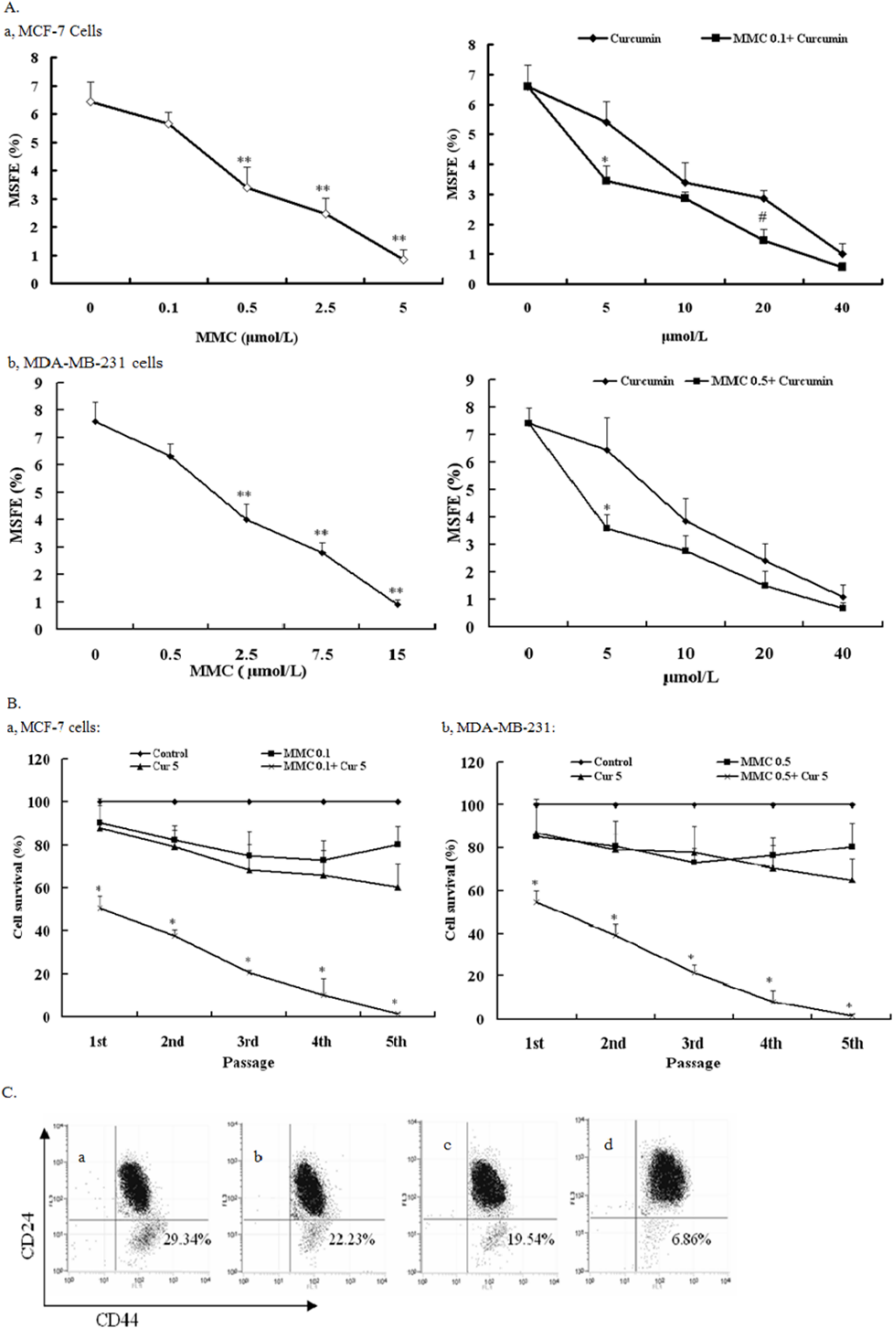
Curcumin and MMC alone and together inhibited mammosphere formation in MCF-7 and MDA-MB-231 BCSCs. (A) MSFE of MCF-7 (a) and MDA-MB-231 (b) BCSCs treated with different doses of curcumin and MMC and their combination was calculated as the percentage of the number of mammosphere (diameter >50 μm) formed in 7 d/original number of cells seeded and is expressed as mean ± SD (n = 3). **P < 0.05, compared with MMC 0 μmol/L. *P < 0.05, compared with curcumin 5 μmol/L. #*P* < 0.05, compared with curcumin 20 μmol/L. (B) Self-renewal capacity of BCSCs was observed in curcumin 5 μmol/L and MMC 0.1 μmol/L alone and together in MCF-7 (a) and both curcumin 5 μmol/L and MMC 0.5 μmol/L alone or together in MDA-MB-231 (b). Data are presented as mean ± SD (n = 3). *P < 0.05, compared with control in different passages. (C) Curcumin sensitizes cancer stem cells and functions synergistically with MMC. Presented are representative flow cytometry dot plots for CD44 and CD24 cell marker expression in MDA-MB-231–derived BCSCs. a, untreated control; b, curcumin treatment; c, MMC treatment; and d, curcumin + MMC treatment.

To determine the effect of curcumin and MMC on the BCSC population, we treated MDA-MB-231- and MCF-7–derived BCSCs in the presence of curcumin only, MMC only, and both curcumin and MMC. The number of mammosphere structures formed revealed that curcumin at 5 μmol /L did not significantly alter the BCSC population in the 2 cell lines (Fig 2A). By contrast, MMC caused a dose-dependent reduction in the MSFE with an IC_50_ of 0.5 μmol/L for MCF-7 and 7.5 μmol/L for MDA-MB-231 cells (Fig 2A). Curcumin at 5 μmol/L and MMC at 0.1 μmol/L and 0.5 μmol/L reduced 50% of the MSFE in MCF-7 and MDA-MB-231 cells, respectively. Thus, a 5- and 15-fold reduction in the MMC concentration was required to achieve a 50% MSFE reduction in the 2 cell lines, respectively.

The effect of MMC on the self-renewal of MDA-MB-231 and MCF-7 cells with and without curcumin was studied. The formation of mammosphere structures revealed that MCF-7– and MDA-MB-231–derived BCSCs propagated when treated with 5 μmol/L curcumin along with 0.1 and 0.5 μmol/L MMC, respectively. However, the ability of BCSCs to propagate decreased gradually with passages and was completely lost at the fifth passage (Fig 2B). During the whole process, cells contained with curcumin and MMC were disaggregated without adding any cells. Because either MMC or curcumin alone did not significantly reduce the propagation of BCSCs with increasing number of passages (Fig 2B), these results suggest that the combination of MMC and curcumin effectively blocked the self-renewal of BCSCs.

In addition, examining the cell surface distribution of CD44 and CD24 through flow cytometry showed that combined curcumin and MMC treatment (and not individually) diminished the BCSC population; this effect was affirmed by the number of CD44^+^CD24^−/low^ cells (29.34% in untreated control and 6.86% in curcumin and MMC cotreated cells; Fig 2C), thus supporting the hypothesis that a combined curcumin and MMC treatment prevents BCSC self-renewal.

### Curcumin improves the tumoricidal effect of MMC on BCSC-derived tumors

**Fig 3:**
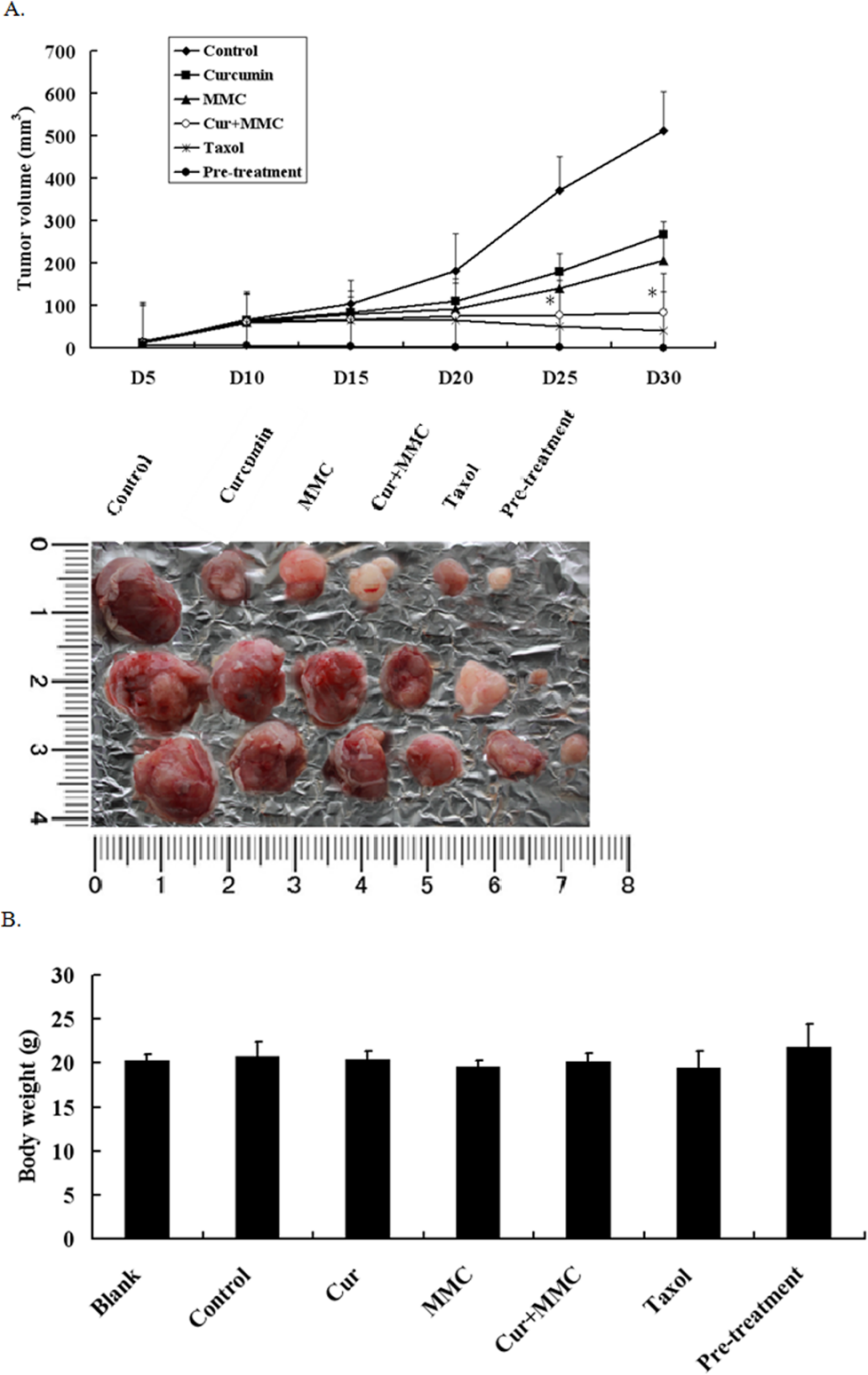
Effect of MMC or MMC+ curcumin on tumor outgrowth in MDA-MB-231 BCSCs xenografts. BALB/c nude mice bearing MDA-MB-231 BCSCs in fat pads as xenografts were treated with daily i.p. injection of control or curcumin and MMC for 4 wk. Tumor volumes (A) and mouse body weights (B) were determined as described. Data are presented as means ± SD (n = 10). *P < 0.05, compared with MMC alone.

The combined ability of MMC and curcumin to effectively block BCSC self-renewal prompted us to investigate whether curcumin also assists chemotherapeutic agents, such as MMC and taxol, to target BCSCs in vivo. To test this possibility, athymic nude mice were inoculated with MDA-MB-231 cell–derived BCSCs. After 1 wk, the animals were injected daily i.p. with 100 mg/kg curcumin and 1.5 mg/kg MMC for 4 wk. When the mice receiving the BCSCs developed sizable tumors, treatment of curcumin or MMC alone reduced 50–60% of the tumor volume in 30 d. By contrast, the combined treatment of curcumin and MMC caused a more than 90% tumor volume reduction in 30 d (Fig 3A). Moreover, none of these treatment regimens exhibited apparent toxicity based on animal body weight (Fig 3B). These results suggest that the combined treatment of curcumin and MMC was more effective in eliminating BCSCs in the primary xenograft than curcumin or MMC alone, further indicating that this regimen can target BCSCs with high potency.

### Effects of verapamil on the reversal of resistance to MMC but not to curcumin

**Fig 4:**
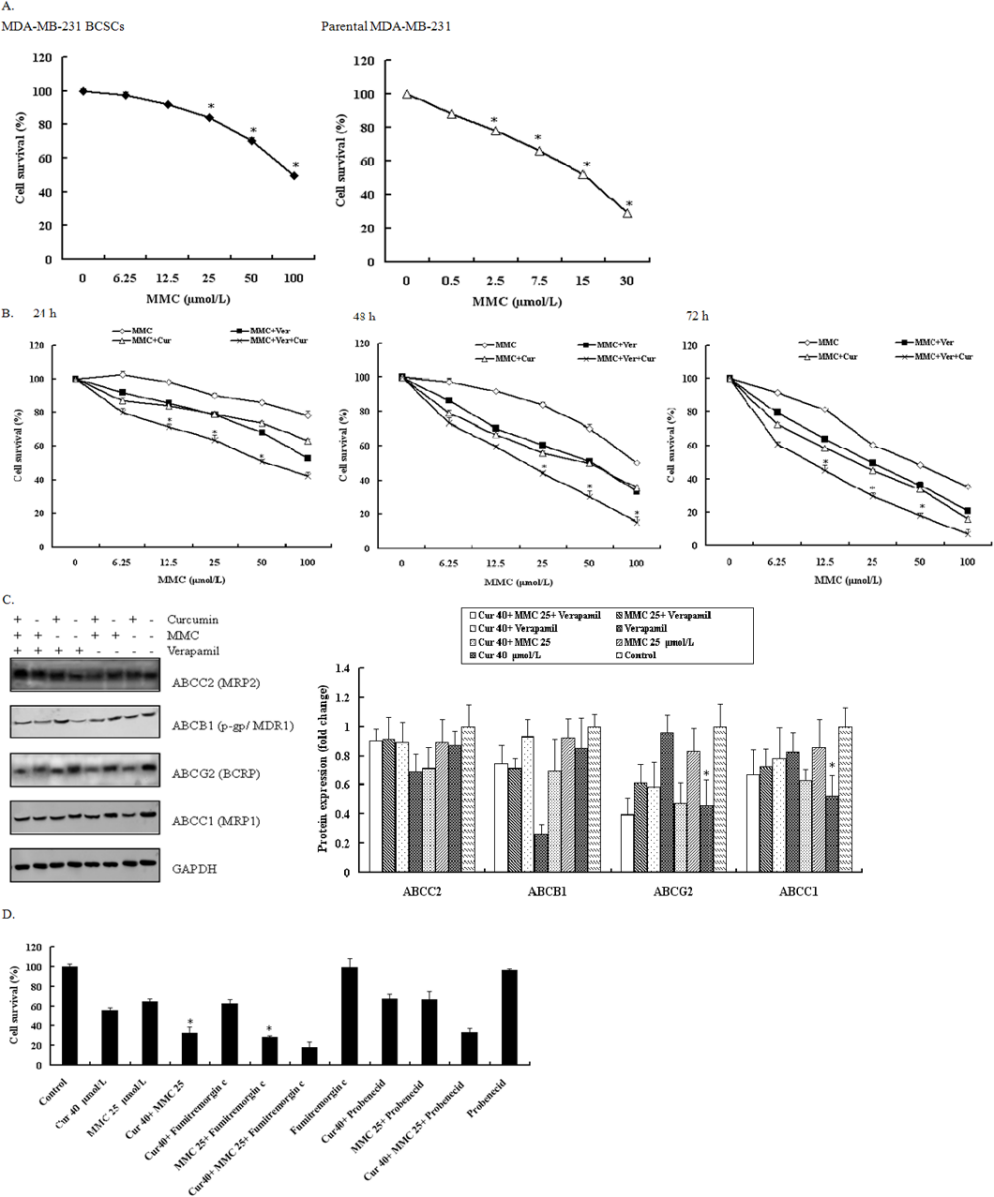
Effect of verapamil and curcumin on reversal of resistance to MMC in MDA-MB-231-derived BCSCs. (A) Cell survival was observed in MDA-MB-23-BCSCs and parental MDA-MB-231. Cells were treated with different concentrations for 48 h. *P < 0.05 compared with untreated control. (B) Dose- and time-dependent effect of MMC with and without verapamil and curcumin. *P < 0.05 compared with MMC and verapamil at corresponding doses. Cell survival was analyzed using MTT. Values are mean ± SD from 3 independent experiments. (C) Western blot analysis of ABCC2, ABCB1, ABCG2, and ABCC1 by curcumin and MMC with and without verapamil treatment in MDA-MB-231 BCSCs. Cells were incubated with 40 μmol/L curcumin, 25 μmol/L MMC, 10 μM/L verapamil and their combinations during 72 h. *P < 0.05 compared with MMC alone. (D) MDA-MB-231-derived BCSCs were treated with 40 μmol/L curcumin and 25 μmol/L MMC alone and together with and without fumitremorgin C and probenecid for 72 h. An MTT assay was performed. Values are mean ± SD from 3 independent experiments. *P < 0.05 compared with MMC alone.

A hallmark of cancer stem cells is the enhanced ABC transporter expression that protects them from cytotoxic agent–induced damage. The observation that curcumin enhances the ability of MMC to induce BCSC cell death led to the hypothesis that curcumin modulated ABC transporter expression in BCSCs. To test this hypothesis, we first determined the IC_50_ of MMC in parental MDA-MB-231 cells and MDA-MB-231–derived BCSCs. An MTT viability assay showed a 6-fold increase in the IC_50_ of MMC in MDA-MB-231–derived BCSCs over their parental cells (Fig 4A). We subsequently examined the IC_50_ of MMC in MDA-MB-231–derived BCSCs with curcumin or verapamil. Verapamil reduced the IC_50_ of MMC from 50 to approximately 30 μmol/L, whereas curcumin reduced the IC_50_ to 20 μmol/L at 72 h (Fig 4B). We subsequently analyzed the effect of curcumin on ABC transporter levels in BCSCs through Western blotting; compared with untreated or MMC-treated cells, curcumin blocked 50% of ABCG2 and ABCC1 (Fig 4C). By contrast, verapamil did not significantly alter the expression of any of these ABC transporters (Fig 4C).

To investigate whether curcumin sensitizes BCSCs by downregulating ABCG2 and ABCC1 expression, we treated MDA-MB-231–derived BCSCs with fumitremorgin C (selective ABCG2 inhibitor) or probenecid (selective ABCC1 inhibitor). Fumitremorgin C, unlike probenecid, substantially decreased the cell viability of MMC-treated cells (Fig 4D), suggesting that curcumin increases MMC-mediated cell death in BCSCs by blocking ABCG2 expression.

## Discussion

Curcumin is a natural compound derived from turmeric (*Curcuma longer*) and exhibits an antitumorigenic effect on various cancers. Curcumin induces apoptosis in breast cancer cells and delays the outgrowth of mammary tumors in neu transgenic mice [29]. The combination of docosahexaenoic acid and curcumin inhibits 7,12-dimethylbenz(a)anthracene-(DMBA)-induced mammary tumorigenesis in mice [30]. In addition, curcumin can reverse multidrug resistance in human colon carcinomas and lung cancer cells in vitro and in vivo [31,32]. We demonstrated that curcumin increased the sensitivity of MDA-MB-231 and MCF-7 cells to chemotherapeutic drugs (paclitaxel, cisplatin, and doxorubicin) (Fig 1). Several studies have suggested that the ability of curcumin to sensitize cancer cells or reverse drug resistance is associated with its effect on the expression or activity of ABC transporters. For example, curcumin inhibits the expression of ABCC1/MRP1 in retinoblastoma cells [33] and suppresses the activity of ABCG2/BCRP in mice [34]. Determining how curcumin affects BCSCs within the mammosphere and tumors is of immense academic interest.

In recent years, cancer stem cells have gained increasing attention as key tumor-initiating cells that may be integral in recurrence following chemotherapy [35]. Cancer stem cells with appropriate intracellular triggers and/or signaling from extracellular environment can purportedly differentiate to initiate tumorigenesis and impart chemoresistance [36]. Fibroblast-derived exosomes [37] and 5-aminolevulinic acid [38] contribute to chemoresistance through cancer stem cell priming.

The cancer stem cell theory states that various cancers are driven and sustained by a small population of cancer stem cells [39]. Moreover, experimental evidence strongly suggests that cancer stem cells can confer drug resistance to tumors [40], causing relapse [41]. However, the effective treatment of resistant and recrudescent tumors is lacking. Targeting the cancer stem cell population is a promising approach to overcome this challenge. Several dietary compounds, such as ginsenoside and pomegranate extract, are currently used as chemopreventing agents against cancer stem cells. In this study, we showed that the dietary compound curcumin substantially improved the tumoricidal effect of MMC on BCSCs. In the presence of curcumin, a 50% MSFE reduction was achieved through a 5- and 15-fold reduction in MMC concentration in MCF-7 and MDA-MB-231 cells, respectively. We showed that BCSCs cultured with a combination of MMC and curcumin had diminished cell numbers with increasing number of passages and were unable to grow beyond the fifth passage. Moreover, the BCSC population revealed that curcumin and MMC in combination diminished the number of CD44^+^CD24^−/low^ cells. Therefore, curcumin decreases the self-renewal of BCSCs in vitro and reduces the BCSC population within the mammosphere (Fig 2).

BCSCs have superior tumor-forming abilities in nude mice compared with their parental cells [42,43]. We showed that a daily injection of curcumin with MMC for 4 wk completely suppressed BCSC-derived tumor growth (Fig 3). These results are consistent with the hypothesis that curcumin effectively suppresses the BCSC population. Two fundamental properties of cancer stem cell–like cells are their ability to self-renew and to generate differentiated progeny [44]. We showed that BCSCs cultured in the presence of MMC and curcumin had diminished cell numbers with increasing number of passages (Fig 2B) and inhibited tumor growth (Fig 3A). Our results indicate that curcumin targets BCSCs by suppressing BCSC self-renewal in vitro and in vivo.

The quiescent nature of cancer stem cells is considered an intrinsic defense mechanism that partially contributes to chemoresistance and tumor recurrence [45,46]. Mammosphere clones exhibit higher chemoresistance than their parental cells do. We showed that the IC_50_ of MMC in MDA-MB-231 or MCF-7 mammospheres was much higher (up to 3- to 4-fold) than that of their parental cells (Fig 4A). The chemoresistance of BCSCs may be due to the overexpression of particular ABC transporters, because verapamil increased the sensitivity of BCSCs to MMC (Fig 4B). We observed that curcumin downregulated ABCG2 and ABCC1 expression (Fig 4C). Additional experiments showed that fumitremorgin C, a selective ABCG2 inhibitor, sensitized BCSCs to MMC to a similar degree as curcumin did (Fig 4D). The clinical neurotoxicity of fumitremorgin C limits their use for cancer therapy. The nontoxic nature of curcumin suggests that curcumin is a potential substitute for fumitremorgin C.

In addition, curcumin also induces apoptosis through mitochondrial pathways involving caspase 8, BID cleavage, cytochrome C release and caspase 3 activation. Bcl-2 and Bcl-xl are critical negative regulators of curcumin-induced apoptosis [47]. Moreover, we have been reported that curcumin combined with MMC induces apoptosis of breast cancer cells [48]. The follow-up study that curcumin improved drug-resistance by inducing apoptosis would be further investigated.

## Conclusions

In summary, we demonstrated that a combined treatment using curcumin and MMC effectively suppresses BCSC population. This study provides strong evidence to support the use of curcumin in chemoprevention.

## Supporting Information

S1 Fig The formation of floating spherical colony-like structures was observed after 2 days culturing in this condition in both cell lines. Representative photographs are on the upper (×100). (A) MCF-7 cells. (B) MDA-MB-231 cells.
(TIF)

S2 Fig MCF-7 and MDA-MB-231 cells grew to different sizes in 1 week mostly. Representative photographs are on the upper (×40). (A) MCF-7 cells. (B) MDA-MB-231 cells.
(TIF)
