# Supplementary figures and images for: Curcumin Improves the Tumoricidal Effect of Mitomycin C by Suppressing ABCG2 Expression in Stem Cell-Like Breast Cancer Cells
Source: PLoS One. 2015 Aug 25;10(8):e0136694. doi: 10.1371/journal.pone.0136694 (PMC4549178; doi:10.1371/journal.pone.0136694)

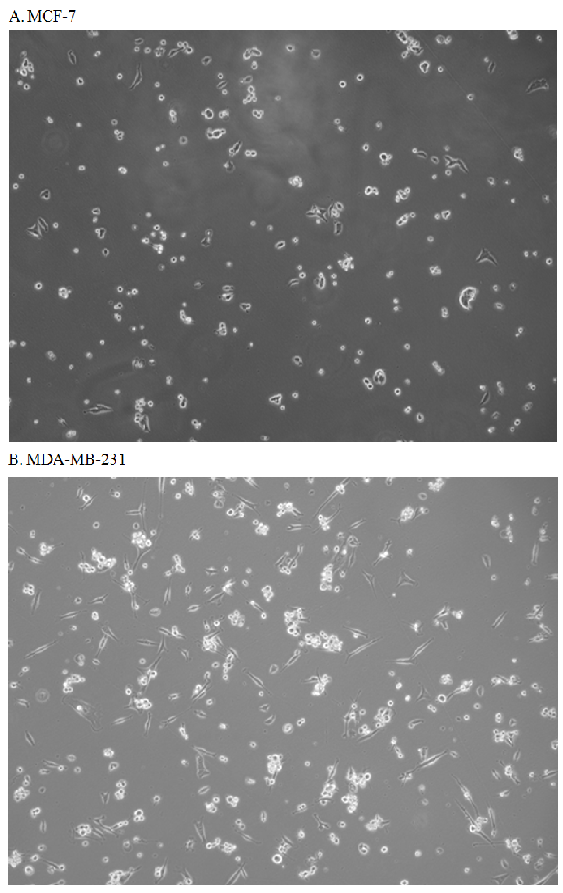

Supplement: S1 Fig — Representative photographs are on the upper (×100). (A) MCF-7 cells. (B) MDA-MB-231 cells. (TIF) [file pone.0136694.s001.TIF]

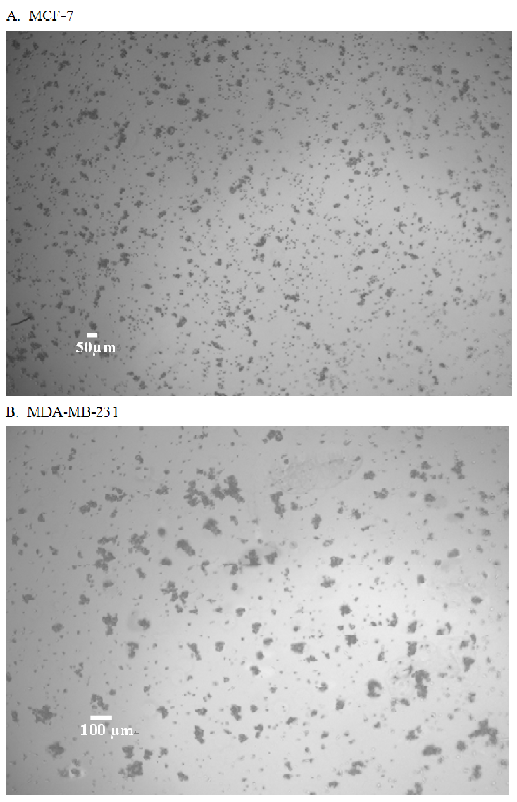

Supplement: S2 Fig — Representative photographs are on the upper (×40). (A) MCF-7 cells. (B) MDA-MB-231 cells. (TIF) [file pone.0136694.s002.TIF]
